# The Microbiome and Irritable Bowel Syndrome – A Review on the Pathophysiology, Current Research and Future Therapy

**DOI:** 10.3389/fmicb.2019.01136

**Published:** 2019-06-10

**Authors:** Pei Pei Chong, Voon Kin Chin, Chung Yeng Looi, Won Fen Wong, Priya Madhavan, Voon Chen Yong

**Affiliations:** ^1^School of Biosciences, Taylor’s University, Subang Jaya, Malaysia; ^2^Department of Medical Microbiology, Faculty of Medicine, University of Malaya, Kuala Lumpur, Malaysia; ^3^School of Medicine, Faculty of Health and Medical Sciences, Taylor’s University, Subang Jaya, Malaysia

**Keywords:** irritable bowel syndrome, microbiome, microbiota dysbiosis, fecal transplant, IBS animal model

## Abstract

Irritable bowel syndrome (IBS) is a functional disorder which affects a large proportion of the population globally. The precise etiology of IBS is still unknown, although consensus understanding proposes IBS to be of multifactorial origin with yet undefined subtypes. Genetic and epigenetic factors, stress-related nervous and endocrine systems, immune dysregulation and the brain-gut axis seem to be contributing factors that predispose individuals to IBS. In addition to food hypersensitivity, toxins and adverse life events, chronic infections and dysbiotic gut microbiota have been suggested to trigger IBS symptoms in tandem with the predisposing factors. This review will summarize the pathophysiology of IBS and the role of gut microbiota in relation to IBS. Current methodologies for microbiome studies in IBS such as genome sequencing, metagenomics, culturomics and animal models will be discussed. The myriad of therapy options such as immunoglobulins (immune-based therapy), probiotics and prebiotics, dietary modifications including FODMAP restriction diet and gluten-free diet, as well as fecal transplantation will be reviewed. Finally this review will highlight future directions in IBS therapy research, including identification of new molecular targets, application of 3-D gut model, gut-on-a-chip and personalized therapy.

## Introduction

Irritable bowel syndrome (IBS) is a common functional gastrointestinal disorder characterized by chronic, recurrent abdominal discomfort and pain, with changes in bowel habits. Patients with IBS can be categorized into four major subtypes depending on the predominant stool pattern, including IBS with constipation (IBS-C), IBS with diarrhea (IBS-D), IBS with mixed bowel habits (IBS-M) and unclassified IBS (Lacy et al., 2016). IBS is frequently encountered in clinical setting, with a prevalence of 10–15% recorded worldwide, despite variations in the criteria used to delineate IBS among countries (Canavan et al., 2014; Sperber et al., 2017). Currently, the Rome IV Diagnostic Criteria, which provides symptom-based criteria is generally applied for diagnosis of IBS and other functional gastrointestinal disorders (Lacy et al., 2016). The impact of IBS on the risk of mortality and socioeconomic status remains elusive, as different epidemiological studies yielded varying results (Canavan et al., 2014). Nevertheless, patients with IBS do account for an increase in health resource utilization and decreased work productivity when compared to healthy individuals (Lembo, 2007).

The onset of IBS-related symptoms often occurrs during adolescence, with females being more susceptible than males in the development of IBS (Canavan et al., 2014). Patients with IBS typically experience abdominal discomfort or pain, and get relief upon defecation, with changes in the stool pattern. Additionally, patients with IBS may experience a range of altered bowel habits, including diarrhea, constipation or alternating constipation and diarrhea. Besides that, digestive symptoms such as dyspepsia, dysphagia, non-cardiac chest pain and nausea are also frequently encountered in patients with IBS. On the other hand, IBS also showed comorbidity with other functional gastrointestinal disorders and association with non-gastrointestinal disorders such as chronic pelvic pain, temporomandibular joint disorder, fibromyalgia and chronic fatigue syndrome (Noddin et al., 2005; Soares, 2014). Most profoundly, psychiatric associated comorbidities such as anxiety, depression and somatoform disorders are highly linked with IBS where these comorbidities require further medical attention. If left untreated, these psychiatric comorbidities will impose negative impact on the quality of life (Bonavita and De Simone, 2008; Soares, 2014).

The human gut is a complex structure and is inhabited by trillions of microorganisms including bacteria, fungi, viruses, eukaryotes, and archae. These vibrant microbial communities are imperative in maintaining gastrointestinal homeostasis (Turnbaugh et al., 2007; Marchesi, 2010). Studies have shown that more than 2000 bacterial species reside in the gut with the majority of these species originating from four main phyla: Bacteroidetes, Firmicutes, Actinobacteria, and Proteobacteria (Li et al., 2014; Hugon et al., 2015). The human gut is highly dynamic and undergoes temporal changes from birth to adulthood. As reviewed by Dominguez-Bello et al. (2011), gut microbiota undergoes a few stages of evolution, whereby its initial composition is strongly determined by mode of delivery (either C-section or vaginal delivery), which will then determine the microbiome profile in later stages of development. Subsequently, the gut microbial community continues to grow and diversify in the first few years of life, stabilizes in the adolescence and starts to decline in the adult. Furthermore, the gut microbiome profiles tend to vary between different geographical regions, populations and development stages, indicating that the gut microbiota is constantly evolving throughout life (Dominguez-Bello et al., 2011).

Recent research suggests that the human gut is likely to be affected by environmental factors including xenobiotics, stress, diet and lifestyle throughout the individual’s lifespan. Furthermore, recent studies also suggest that gut microbiome is likely a good predictor for metabolic variables and clinical phenotypes and is an important key player in IBS pathogenesis (Rothschild et al., 2018). Hence, this review aims to expound the pathophysiology of IBS and the influence of gut microbiome on IBS pathogenesis, and shed light on current advances and future directions in IBS research and therapeutic interventions.

## Methods

The content and information of this review were based on literature published in PubMed, Scopus, and JCR-ISI. This article does not involve any human or animal experimental studies. Articles published before August 2018 in the aforementioned databases were included. A topic-centric search was performed for each particular section in order to explain and describe the topic. The relevant articles related to the section were identified and the bibliographies were used to perform recursive search to obtain original as well as additional references. The search terms included “irritable bowel syndrome,” “microbiota,” “microbiome,” and “IBS treatment.” These search items were combined with the AND operator to additional search terms for the relevant sections of the review, including “pathophysiology,” “animal studies,” “metagenomics,” “prebiotics,” “probiotics,” “fecal transplantation,” “symbiotic,” “postbiotic,” “metagenome,” “fungal treatment,” “archaebiotics,” “phage therapy,” “3-D gut modeling,” “dietary intervention,” “pathogenesis,” “FODMAP,” “diet,” “meta-analysis,” and “future therapy.” All authors contributed toward the literature search.

## Pathophysiology of IBS

**FIGURE 1:**
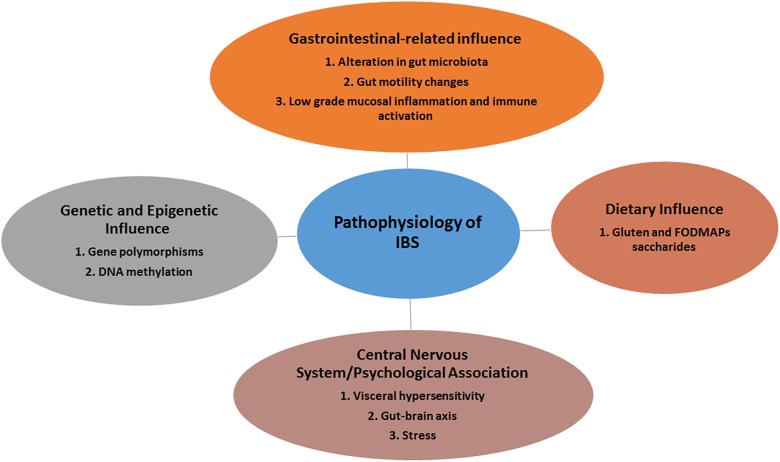
The various players involved in the development of IBS.

The pathophysiology of IBS remains poorly understood. Though non-specific, certain pathogenic factors including genetic predisposition, visceral hypersensitivity, food intolerance, altered gut-brain axis and gut dysmotility, dysfunction of innate immunity and dysbiosis may contribute to this disorder (Bellini et al., 2014) (Figure 1). It remains unclear which among these pathogenic factors trigger or augment the IBS as symptoms vary among different individuals.

### Genetic Influence

Accumulating evidences have shown that genetic risk in IBS ranges from complex polygenic cases with mixtures of common variants to rare single gene aberration (D’Amato, 2013). The discovery of mutation in the *SC5NA* gene encoding a sodium channel ion, which is associated with abdominal pain experienced by IBS patients, was a notable example of the ability of gene aberration to induce IBS clinical symptoms (Beyder et al., 2014). Subsequently, a number of single nucleotide polymorphisms (SNPs) studies have identified polymorphisms in genes associated with IBS pathogenesis including genes coding for serotonin signaling (Jun et al., 2011; Grasberger et al., 2013), immune regulation and epithelial barrier function (Wouters et al., 2013), bile acid synthesis (Wong et al., 2012) and cannabinoid receptors (Camilleri et al., 2013). Findings from a GWAS in 2015 also identified *GRID2IP* [glutamate receptor, ionotropic, delta 2 (Grid2) interacting protein] and *KDELR2* (KDEL endoplasmic reticulum protein retention receptor 2) to be linked to risk of IBS development (Ek et al., 2015). Meanwhile, report from another GWAS could not confirm the dominant roles for most of the SNPs in immune-related genes in IBS development, except for SNPs in *TNFSF15* (Czogalla et al., 2015). Overall, the impact of genetic influence on IBS development remains obscure due to relatively small cohort studies and the absence of prominent structural abnormalities. The susceptibility of common and rare gene variants in IBS remains largely unknown. Additionally, epigenetic factors such as DNA methylation could manifest in IBS (Mahurkar et al., 2016). Hence, further inspection on gene-gene interactions, gene-environment interactions, and gene-pathways interactions are warranted and are more likely to give us clues in understanding IBS pathogenesis.

### Gastrointestinal Associated IBS Pathophysiology

#### Alteration in Gut Microbiota

Under normal circumstances, mucus epithelium barrier confines microbes to the epithelial surface or intestinal lumen where homeostatic immune responses are induced to maintain barrier integrity and tolerance among commensal microbes. This enables microbes to persistently colonize the intestine and perform symbiotic functions. However, once the barrier is breached by influx of inflammatory mediators, pathogens or any agents that provoke intense immune reactions, severe inflammation occurs and this will affect the intestinal environment, and changes the gut microbiota composition (Pedron and Sansonetti, 2008). Briefly, alteration in gut microbiota could contribute to IBS pathogenesis by altering gut immunity and integrity, and modulation of gut neuromuscular junction and gut-brain axis. Multiple reports have linked IBS pathogenesis with dysbiosis, a condition that refers to decrease/loss of microbial diversity and richness, owing to the changes from commensal bacteria to pathogens in the human gut (Carroll et al., 2011, 2012). For example, the composition and activities of *Lactobacilli* and *Bifidobacteria* are heavily compromised in IBS patients (Bellini et al., 2014). A recent study also identified a specific intestinal microbiota signature that could be linked to the severity of IBS (Tap et al., 2017). In this study, the authors reported that the severity of IBS was positively correlated with low CH_4_ exhaled, low microbial richness, absence of *Methanobacteriales* and enrichment with *Bacteroides* enterotypes. On the other hand, growing evidence of the involvement of mycobiome alterations in IBS patients and the development of visceral hypersensitivity indicates fungi dysbiosis may have indispensable role in IBS pathogenesis (Botschuijver et al., 2017). The beneficial effects of probiotics on alleviating visceral sensitivity, intestinal permeability and inflammation further support the role of gut microbiota in IBS (Ohman and Simrén, 2013).

#### Low Grade Mucosal Inflammation and Immune Activation

Recent studies have linked IBS pathogenesis with low grade mucosal inflammation. Combination of low grade mucosal inflammation with visceral hypersensitivity and impaired bowel motility could be the underlying etiology for IBS pathogenesis. This condition may arise from compromised epithelial barrier (Piche et al., 2009), post-infectious alterations (Beatty et al., 2014), dysbiosis (Simrén et al., 2013), and altered stress levels (Qin et al., 2014), which stimulate aberrant immune responses. Associated mucosal inflammation in IBS patients is often linked with history of infectious gastroenteritis induced by bacteria, parasites or viruses, which is referred to as post-infectious IBS (PI-IBS) (Beatty et al., 2014). This association is further consolidated by findings from several studies including a meta-analysis which demonstrated an approximately sevenfold of increased risk of developing PI-IBS (Halvorson et al., 2006). A number of risk factors have been identified for the development of PI-IBS, including young age, female gender, depression, anxiety and prolonged initial infection with fever (Thabane et al., 2007). Additionally, alteration in immune responses due to inflammation provoked by dysbiotic microbiota, increased number of immune cells such as mast cells and lymphocytes seen in the intestinal mucosal biopsies from PI-IBS patients, and increased cytokine production (Jalanka-Tuovinen et al., 2014; Sundin et al., 2014; Downs et al., 2017) further suggest the pivotal role of post infectious syndrome in IBS development.

A substantial number of studies also documented the role of innate immune dysfunction in IBS pathogenesis and its impact on low-grade inflammation, at both systemic and mucosal level. Activation of immune system in colonic mucosa followed by infiltration of numerous immune cells and release of inflammatory cytokines are observed in IBS patients as compared to healthy subjects (Martin-Viñas and Quigley, 2016; Lazaridis and Germanidis, 2018). Additionally, elevated pro-inflammatory cytokines in isolated PBMCs from patients with IBS-D, particularly IL-6, TNF-α, and IL-1β were observed. These secreted cytokines are also highly associated with depression and anxiety, suggesting the role of gut in regulating brain function (Liebregts et al., 2007). A recent study highlighted the association of inflammatory cytokines IL-17 and TNF-α with disease symptoms and quality of life (IBS-QoL) in different subtypes of IBS patients (Choghakhori et al., 2017). The underlying cause for this altered immune response remains unclear. Nevertheless, impairment in the integrity of mucosal epithelial barrier is likely to be the cause for this condition. Studies in IBS patients with and without infective etiology also showed increased levels of intestinal permeability, suggesting that altered intestinal permeability aroused from defective mucosal epithelial barrier might interfere with gut immune homeostasis, which subsequently promote gut inflammation and aberrant immune responses (Marshall et al., 2004; Shulman et al., 2014).

Currently, most of the studies had focused on specific immune cells, pathogen recognition receptors and cytokines independently, which have yielded varying results and failed to depict the precise role of innate immune dysfunction in the pathophysiology of IBS. More comprehensive approaches should be incorporated to elucidate specific immune signaling pathways involved and the interaction between gut microbiota, enteric nervous system and immune activation during IBS pathogenesis (Lazaridis and Germanidis, 2018). Further examination of immune responses from biopsy samples might provide useful information in elucidating the IBS pathophysiology and identify any potential disease indicators (Choghakhori et al., 2017). Consequently, specific therapeutic interventions can be designed to help combat or mitigate the burden of this disease.

#### Gut Motility Changes

Patients with IBS often have changes in their gut motility, which is usually driven and augmented by stress via gut-brain axis (Drossman, 2016). These changes are observed in patients with diarrhea, constipation or both and is likely influenced by alteration in serotonin (5-HT) metabolism. Serotonin has a prominent role in controlling gastrointestinal motility. High serotonin levels were observed in patients with diarrhea predominant IBS while low levels of serotonin is associated with constipation predominant IBS (Crowell, 2004; Camilleri, 2009).

### Dietary Influence

Diet plays a crucial role in IBS pathogenesis through modulating the normal gut microenvironment including decrease in colonic fermentation, altered gut microbiome composition and reduced antigen activation by the gut immune system (Muegge et al., 2011; David et al., 2014). Growing evidence has supported the importance of diet in IBS etiopathogenesis. Food and breakdown products of food can affect many aspects of gut physiology including motility, permeability, microbiome, visceral sensation, brain-gut interactions, immune regulation and neuro-endocrine function; which are all relevant to the pathogenesis of IBS (Chey, 2016). Thus, a diet that limits intake of “offending” foods that trigger these alterations, or a diet that corrects the microbiota dysbiosis, would be beneficial to manage IBS symptoms.

Exacerbation of symptoms with ingestion of specific foods such as gluten and FODMAPs saccharides are often reported in patients with IBS. Abdominal pain and other GI-related symptoms were reported in IBS patients after consumption of gluten, without the clear evidence of celiac disease based on histology and serology assessments. This condition, so called “non-celiac gluten sensitivity (NCGS)” may be pertinent in IBS-like symptoms development (Elli et al., 2015). The effects of gluten on IBS most likely include alteration of intestinal permeability and activation of autonomous and enteric nervous system (Biesiekierski et al., 2013; Elli et al., 2015).

Ingestion of FODMAPs have been shown to lead to bloating, abdominal pain and other IBS symptoms in about 70% of patients (Staudacher et al., 2012; Böhn et al., 2015). FODMAPs are short-chain carbohydrates that are easily fermentable by gut bacteria into methane and hydrogen gasses but are poorly absorbed (Eswaran et al., 2016). Short chain fatty acids are also another byproduct of FODMAPs fermentation. The gasses produced by FODMAPs lead to bloating symptoms in IBS. The osmotic effects of FODMAPs also increase intraluminal fluid which may cause GI distension and stimulate abnormal intestinal motility.

### Central Nervous System/Psychological Association in IBS Pathophysiology

#### Visceral Hypersensitivity

Visceral hypersensitivity, a condition of increased pain sensation in the bowel due to physiological stimuli is crucial in IBS pathogenesis. Patients with visceral hypersensitivity tend to have lower colonic distension pain threshold where a normal stimulus will intensify the pain in the patients (Camilleri et al., 2001). Visceral hypersensitivity may occur at both peripheral and central nervous system and is the key pathogenesis for IBS (Aziz et al., 2007). Epidemiological data from various studies reveal that prevalence of visceral hypersensitivity in IBS patients varied from 33 to 90%, with a higher tendency to develop in IBS-D patients (Kanazawa et al., 2008; Gwee et al., 2009; Ludidi et al., 2014). Additionally, clinical evidence also showed that IBS patients with visceral hypersensitivity developed more severe complications than those without visceral hypersensitivity (Ludidi et al., 2014). The pathophysiology of visceral hypersensitivity in IBS remains poorly understood. Multiple factors are involved in this altered visceral sensation including dysbiosis, brain–gut communication, diet, psychological factors, genetic predisposition, inflammation and immunological factors. Additionally, altered intestinal permeability may contribute toward visceral sensitization and severity of IBS complications (Farzaei et al., 2016). Findings from clinical studies also revealed high expression of circulating microRNAs (miRNAs) in the colonic tissues of IBS patients, suggesting the involvement of epigenetic and genetic events in modulating intestinal signaling pathways, particularly serotonin receptor gene with a *cis*-regulatory variant on the somatic hypersensitivity and intestinal permeability of IBS patients (Kapeller et al., 2008). On the other hand, sensitization of distal peripheral afferent, particularly serosal and mesenteric afferents within the splanchnic pathway could be the underlying factors for visceral hypersensitivity in IBS patients. These distal peripheral afferents possess chemosensitivity which could prolong visceral sensitization and induce post-inflammatory effects (Anand et al., 2007). Hence, targeting microbiota-gut-brain and local neuroimmune pathways could be potential therapeutic intervention in managing visceral hypersensitivity in IBS patients.

#### Alteration in Gut-Brain Axis

The brain-gut axis (GBA) is a bidirectional communication system between the gut and the brain. Along this conduit, the brain interacts with the gut through neural components (CNS and ANS), endocrine system (hypothalamic-pituitary-adrenal axis), immune components (cytokines and metabolic) and gastrointestinal components (microbiota, intestinal barrier and intestinal immune response) (Oświęcimska et al., 2017). Studies have shown that disturbances in GBA drive the pathogenesis in neurodegenerative disorders (Cenit et al., 2017) and gastrointestinal diseases including inflammatory bowel syndromes and inflammatory bowel diseases (IBDs) (Bonaz and Bernstein, 2013; Oświęcimska et al., 2017).

The most notable IBS comorbid disorders, anxiety and depression are highly seen in community samples and outpatients. However, the linkage between these disorders and IBS patients were not fully elucidated by healthcare seeking behavior alone. Hence, it was postulated that the brain drives these psychiatric comorbidities seen in IBS patients, which leads to the conclusion that IBS is a primary brain disorder or somatic symptom disorder whereby the brain is involved in manifesting the gastrointestinal symptoms (Henningsen et al., 2003; Tanaka et al., 2011; Patel et al., 2015). Nevertheless, several prospective studies reported that at least in half of the IBS cases, gastrointestinal symptoms appeared first, followed by mood disorders (Jones et al., 2012; Koloski et al., 2012, 2016). In another independent study, incidence of mood and anxiety disorders after the onset of IBS were around 40 and 23% respectively. These findings likely suggest that the gut is driving the brain manifestations (Sykes et al., 2003). Additionally, studies related with gut microbiome, intestinal inflammation and immune response further suggest the concept that the gut drives brain alterations (Liebregts et al., 2007; Mayer et al., 2014). Eventually, if these findings are ultimately accurate, reversing gastrointestinal dysfunction could serve as potential therapeutic interventions in curing IBS and other concomitant mood disorders.

#### Stress

On the other hand, psychological stress may have critical influence on gut-brain axis. IBS is considered as a stress sensitive disorder. The main effects of stress are mainly on intestinal motility and permeability, visceral sensitivity, immune responses and gut microbiota composition (Qin et al., 2014). The underlying mechanism is likely through secretion of pro-inflammatory cytokines such as interleukin-6 (IL-6) and interleukin-8 (IL-8) which activate hypothalamic-pituitary-adrenal (HPA) and hypothalamic-autonomic nervous system axes, trigger the release of corticotrophin releasing factor (CRF), adrenocorticotropic hormone and cortisol, which consequently affect gut homeostasis (Dinan et al., 2006). The influence of stress on IBS is indisputable. Thus, managing stress and stress-induced responses are imperative as part of the therapeutic intervention in IBS patients. Other psychological factors including abuse (physical, sexual, verbal, and adverse life events), posttraumatic stress disorders and somatization are also likely involved in gut–brain interaction and IBS pathogenesis (Qin et al., 2014).

## Microbiota and Microbiome in IBS

### Gut Microbiota Composition in IBS

The human microbiota in the intestines is a complex assemblage of microbes, a dynamic environment comprising pro- and anti-inflammatory bacterial species (Dupont, 2014). Apart from bacteria, intestinal microorganisms such as archae, fungi and viruses exist in symbiosis in healthy individuals. Most bacteria in the gastrointestinal tract include members from the following phyla in a descending order: Firmicutes (64%), Bacteroidetes (23%), Proteobacteria (8%), and Actinobacteria (3%) (Gill et al., 2006; Bäckhed et al., 2012). However, only about one third from the ∼1000 different bacterial species had been identified and characterized so far. Many of the species from Archae that had been isolated are methanogens and halophilic archae. The methanogens associated with gut include *Methanobrevibacter smithii* (94%), *M. stadtmanae* (23%), *Candidatus* “*Methanomethylophilus alvus*” and *Candidatus* “*Methanomassiliicoccus intestinalis*,” among others (Dridi et al., 2009). As polysaccharides get fermented by gut bacteria, gasses such as hydrogen (H_2_) and methane (CH_4_) are generated as by-products (Pimentel et al., 2006). In addition, short-chain fatty acids (SCFAs) such as acetate, propionate and butyrate are also produced by the colonic bacteria whereby these SCFAs and gasses could affect bowel movement as well as gut permeability. One study by Pozuelo et al. (2015) found a lower abundance of butyrate-producing bacteria in IBS patients, particularly in IBS-D and IBS-M. As butyrate-producing bacteria are known to improve intestinal barrier function, decreased amount of these bacteria in IBS-D and IBS-M patients could have led to impairment in intestinal permeability and activate the nociceptive sensory pathways, which manifest in the symptoms observed. They also found that IBS patients not undergoing any therapy had lower abundance of Methanobacteria (Pozuelo et al., 2015). This observation was in agreement with several other studies which found that methane production was seen at lower levels in IBS-D and higher levels in IBS-C (Jahng et al., 2012; Tap et al., 2017). Besides the sulfate-reducing bacteria, methanogens from the Archaea kingdom are the main gut microbe responsible for removing excess hydrogen by converting it to methane. Methane production capacity is linked to low transit time and anti-inflammatory effects in the colon. In IBS patients, lower counts of methanogens imply reduced ability for hydrogen gas removal from the colon, and hence this could attribute to flatulence or excess gas in the abdomen.

Among the common trends reported in previous studies on the composition of intestinal bacteria in IBS subjects relative to healthy controls were increase in abundance of Proteobacteria, including from the phyla *Veillonella*, and Firmicutes, including *Lactobacillus* and *Ruminococcus* (Tana et al., 2010; Rigsbee et al., 2012). This was usually accompanied by a decreased quantity of *Bifidobacterium*, *Faecalibacterium, Erysipelotrichaceae* and methanogens (Rajilić-Stojanović et al., 2011; Pozuelo et al., 2015). The halophilic archae such as *Halorubrum koreense*, *Halorubrum alimentarium*, *Halorubrum saccharovorum*, and *Halococcus morrhuae* were isolated from a study on Korean subjects (Nam et al., 2008). This could also be due to Koreans’ high-salt food intake, which warrant further studies on the prevalence of these halophilic archae in relation to diet. Out of the 62 fungal genera that were isolated from 98 healthy individuals in the US, the predominant fungi were *Saccharomyces* (present in 89% of the samples), followed by *Candida* (57%) and *Cladosporium* (42%) (Hoffmann et al., 2013). However, thus far gut microbiome studies had reported the distribution of total fungi present as part of the microbiome as only 0.1% (Underhill and Iliev, 2014; Tang et al., 2015). It is possible that this could be an under-representation due to depth limitations of current metagenomics techniques, where sequences of dominant microbes will “crowd out” the detection of less abundant fungi.

### Microbiome – From Symbiosis to Dysbiosis and Pathogenesis

The gastrointestinal microbial flora inhabits in the human host through an intriguing relationship of symbiosis or “commensalisms.” This delicate symbiosis starts at birth when the infant crosses the birth canal and develops in a regulated and coordinated manner as the infant matures and develops into adulthood. It is widely believed that the GI microbiota in an infant is derived from its mother’s microbes during either vaginal delivery or cesarean section and possesses low complexity as well as species diversity. As the infant grows, the microbiota also dynamically matures and gradually attains richness in diversity that approximates the adult microbiota profile by the first year of life. Distinct life events, such as weaning from breast milk to solid foods are postulated to be the cause of the increase in species diversity. It has been postulated that the presence of a microbial flora that co-exist within the individual host in well-established symbiosis may act as a deterrent for pathogenic microbes from colonizing and disrupting the resident microbiota.

Dysbiosis is a condition of having imbalance in the microbial community in or on the body and is sometimes known as impaired microbiota. In a study conducted in Sweden, Norway, Denmark, and Spain, on patients between 17 and 76 years old (Casén et al., 2015), dysbiosis was investigated in 236 IBS and 135 IBD patients who were diagnosed according to Rome II and III-criteria. A dysbiosis frequency of 73% was observed among IBS patients. Dysbiosis in healthy individuals occur at a rate of around 16% (Karantanos et al., 2010; Jeffery et al., 2012; Collins, 2014). In the Casén et al. (2015) study, pre-dominant bacteria contributing to dysbiosis in IBS include Firmicutes such as *Bacillus* and *Ruminococcus gnavus*; Proteobacteria such as *Shigella* or *Escherichia*; and Actinobacteria. *Bacteroides stercoris* and *Bifidobacterium* were also known to contribute to dysbiosis among this patient cohort, which could be affected by the dietary differences between the Scandinavian countries and the Mediterranean region.

A meta-analysis which was performed on 13 studies up until 2016 had shown that there were significant differences in expression in IBS patients compared to healthy controls for *Lactobacillus*, *Bifidobacterium*, and *Faecalibacterium prausnitzii*. However, no significant dysbiosis was shown among the Bacteroides–Prevotella group, *Enterococcus*, *Escherichia coli*, *Clostridum coccoides* and other genera or species among these patients (Liu et al., 2017). Patients involved in this meta-analysis were from China, Finland, France, India, Japan, Netherlands, and United States. Further analysis revealed that IBS-D patients had significant decrease of *Lactobacillus* and *Bifidobacterium*, as compared to their healthy counterparts and constipated-predominant IBS patients. Among Chinese IBS patients, it was reported that *Lactobacilli* showed significant reduction, contrary to patients from other countries (Liu et al., 2017; Zhuang et al., 2017). Therefore, further investigations are required in the case of *Lactobacilli* to identify whether it can survive and adapt well to the pro-inflammatory gut environment or is a contributor to IBS. Increase of *Pseudomonas aeruginosa* was found in two studies indicating a relationship between this bacterium with IBS (Kerckhoffs et al., 2011; Shukla et al., 2015). Other studies reported that increased levels of Bacteroidetes were from the family Enterobacteriaceae such as *Ruminococcus* sp., *Lactobacilli* sp., and *Clostridium* sp. in the IBS patients (Si et al., 2004; Malinen et al., 2010; Ponnusamy et al., 2011). Another organism related to post-infection in IBS patients is the parasite, *Giardia duodenalis* (Beatty et al., 2017). From experiments involving *G. duodenalis* and bacterial biofilms, it was observed that the thickness of the microbiota biofilm was reduced from 100–210 μM to 10–105 μM in the presence of this parasite (Beatty et al., 2017). Further analysis revealed that extracellular matrix (ECM) compositions of the mucosal microbiota biofilms were also altered, along with the structural integrity of the biofilm, with an over-representation of *Clostridiales* bacteria and a decreased amount of *Phascolarctobacterium* sp. Studies have shown that *G. duodenalis*-infected IBS patients from Italy and Norway had disruptions in their mucosal microbiota, leading to chronic post-infections.

Repeated observations from various studies for bacterial dysbiosis in IBS are generally increased or decreased levels of Firmicutes and Bacteriodetes with ratios that differ between study groups. The dysbiosis would also lead to disruptions in the immune function, which contributes to post-infections in IBS patients which could become chronic. A study by Wadhwa et al. (2016) found that around 25% of *Clostridium difficile* infected patients developed IBS, particularly mixed-IBS, 6 months or more post-infection. When compared to healthy individuals, a few studies have demonstrated that there were increased levels of C-reactive proteins, inflammatory mediators such as IL-6 and IL-8 and inflammatory cytokines in IBS patients (Matricon et al., 2012; Ringel and Maharshak, 2013; Hod et al., 2016). Apart from these, selective inhibition of mast cells was also suggested with the presence of bacterial lipopolysaccharides (Ludidi et al., 2015). Some reports have also indicated an increase of *Streptococcus* spp., a pathogenic bacterium which causes increased levels of IL-6, and mucin degraders such as *Ruminococcus* spp. (Rajilić-Stojanović et al., 2011). In some patients, an increased expression of Toll-like receptor (TLR)-4 which recognizes bacterial LPS, TLR-5 which recognizes bacterial flagellin and increased levels of anti-flagellin antibodies were found in post-infection IBS (Anitha et al., 2012).

There had been many instances when commensals (normal microbiota) reportedly turned pathogenic such as the case of *Enterococcus faecalis* which had inadvertently acquired a number of virulence genes that conferred vancomycin resistance (Paulsen et al., 2003). In another study, activation of latent virulence genes in non-invasive *E. coli* was found to be due to the exposure to *C. jejuni* secretory-excretory products (Reti et al., 2015). Sharing such genes with their co-inhabitants may allow adaptation for better adhesions, acquiring new nutritional pathways and antibiotic resistance, all of which lead to evasion from the immune system as a possible pathogenesis step. In the acute phase of giardiasis, dysbiosis has also been indicated to contribute to CD8 T lymphocyte-mediated impairment (Keselman et al., 2016). Clearly, there is a robust connection between bacteria dysbiosis and the severity of IBS. Nonetheless, the relative abundance of the microbiota in different IBS-subtypes remains largely unexplored. Hence, we summarized the microbiota diversity in different IBS-subtypes in this section, as indicated in Table 1.

**Table 1:** Alterations in microbiota diversity in different subtypes of IBS.

IBS subtypes	Microbiota diversity (Family/Phylum/Genus/Species)	Alteration in microbiota (compared with healthy subjects)	References
IBS-C	*Veillonella* spp	Increased	Malinen et al., 2005
	*Lactobacilli* spp.	Increased	
	*R. bromii-*like phylotype	Increased	Lyra et al., 2009
	*B. catenulatum*	Decreased	Kerckhoffs et al., 2009
	*Methanobrevibacter*	Decreased	Rajilić-Stojanović et al., 2011
	*Methanobrevibacter smithii*	Increased	Kim G. et al., 2012
	Unknown Ruminococcaceae, unknown Christensenellaceae, *Akkermansia*, and *Methanobrevibacter*	Increased Increased Increased	Pozuelo et al., 2015
	*Clostridiales*	Increased	Tap et al., 2017
	*Bacteroides*	Decreased	
	*Prevotella*	Decreased	
IBS-D	*Lactobacillus* spp.	Decreased	Malinen et al., 2005
	*Clostridium symbiosum*-like	Decreased	Rajilić-Stojanović, 2007
	*Proteobacteria*	Increased	Krogius-Kurikka et al., 2009
	*Firmicutes* (*Lachnospiraceae*)	Increased	
	*Actinobacteria*	Decreased	
	*Bacteroidetes*	Decreased	
	*B. catenulatum*	Decreased	Kerckhoffs et al., 2009
	*C. thermosuccinogenes*	85% phylotype increased	Lyra et al., 2009
	*R. torques*	94% phylotype increased	
	*Collinsella aerofaciens*	Decreased	
	*B. intestinalis*-like phylotype	Decreased	
	*Lactobacillus* spp.	Increased	Carroll et al., 2010
	*Enterobacteriaceae*	Increased	Carroll et al., 2012
	*Fecalibacterium (Faecalibacterium prausnitzii)*	Decreased	
	Bifidobacteria	Decreased	Parkes et al., 2012
	Ruminococcaceae, unknown Clostridiales, Erysipelotrichaceae, Methanobacteriaceae	Decreased	Pozuelo et al., 2015
	*Clostridiales*	Increased	Tap et al., 2017
	*Bacteroides*	Increased	
	*Prevotella*	Decreased	
	*Lachnospira*	Decreased	Zhuang et al., 2018
	*Parasutterella*	Decreased	
	*Lachnospiraceae_UCG-010*	Decreased	
	*Ruminococcaceae_UCG-003*	Decreased	
	*Lactobacillus*	Decreased	
	*Turicibacter*	Decreased	
	*Enterococcus*	Decreased	
	*Weissella*	Decreased	
	*Oxalobacter*	Decreased	
	*Oceanobacillus*	Decreased	
	*Lachnospiraceae_NK4A136_group*	Decreased	
	*Faecalitalea*	Increased	
	*Prevotella*	Increased	Su et al., 2018
	*Bacteroides*	Decreased	
	*Bifidobacteria*	Decreased	
	*Lactobacillus*	Decreased	
	*Faecalibacterium*	Decreased	Maharshak et al. (2018)
	*Dorea*	Increased	
IBS-A	*C. symbiosum*	Decreased	Rajilić-Stojanović, 2007
	*Prevotella oralis*	Decreased	
	*Ruminococcus torques* (*R. torques* 93% phylotype)	Increased	Lyra et al., 2009
	*B. intestinalis*-like phylotype	Highest	
	*R. torques* 93 %	Decreased	
	*C. cocleatum* 88%	Increased	
	*B. catenulatum*	Decreased	Kerckhoffs et al., 2009
	*Veillonella*	Increased	Tana et al., 2010
	*Faecalibacterium* spp.	Increased	Rajilić-Stojanović et al. (2011)
	Erysipelotrichaceae	Decreased	Pozuelo et al., 2015
	*Clostridiales*	Increased	Tap et al., 2017
	*Bacteroides*	Increased	
	*Prevotella*	Decreased	


## Methodologies for Studying IBS and Associated Microbiome

In this section, we review several methodologies available for studying IBS, including the establishment of animal models, and *in vitro* models with the latest innovations of gut-on-a-chip systems. The importance of fecal sample collection for assessing the intestinal microbiota composition and other associated applications are also highlighted. The technological platforms for studying the microbiome (sets of genes), the metagenome and metabolome, along with high-throughput culture (culturomics) are also presented.

### Experimental Animal Models for IBS

#### The Wrap Restraint Stress (WRS)

Currently, there is no single animal model that can best mimic the human IBS. The WRS model which involves immobilizing the animal for a minimum duration of 2 h, is commonly applied in acute tests. This model often demonstrates morphological changes of the entire colonic wall that shows visceral hypersensitivity similar to human IBS symptoms (Gue et al., 1997). The WRS model is established on the premise that psychosocial stress have an indispensable role in the etiology of IBS. Experimental results show that dysmotility and hypersensitivity in these animals could have resulted from alterations in glial cells and both excitatory and inhibitory neurotransmitters (Traini et al., 2016).

#### Chemical Irritation

Abdominal pain is the main symptom present in most IBS patients (Bray et al., 2006). Animal models of visceral pain are crucial for aiding researchers in unraveling the possible mechanisms that contribute toward IBS etiopathology. The abdominal pain-related behavioral model can be induced by administration of neostigmine, mustard oil, capsaicin and acetic acid solution (Fichna et al., 2012). Pain behaviors in the animals which are measurable include specific postures like licking of the abdomen, forced pressure onto the abdomen and abdominal contractions. Chemical irritation models are useful to study abdominal pain-related signaling pathways in IBS as evidenced by the blockage of pain-related responses in animal by morphine. This model is also frequently applied in drug discovery and therapeutics development for IBS. For example, Chen et al. (2015) showed that berberine can improve intestinal motility and visceral pain in IBS via the opioid receptor dependent manner using chemical irritation method in mice.

#### Chronic Stressors

Apart from acute stress, there is evidence suggesting that IBS may be associated with childhood traumas of varying root causes or the presence of recurring stress in adulthood (Mayer et al., 2015), indicating prolonged stress may be involved in the development and maintenance of IBS. These clinical data prompted scientists to develop IBS animal models with chronic as opposed to acute stressors. The maternal separation (MS) model and the water avoidance stress (WAS) model are two animal models of chronic stress which were established to mimic infancy trauma, and stress in adulthood, respectively. The symptoms produced by these two behavioral animal models resemble human IBS with WAS being considered as a milder chronic stress compared to MS. Interestingly, these models show that there is a gender-difference in response to stress.

#### Maternal Separation

In this model, young puppies were removed from the mother for a few hours per day during the first 2 weeks of life. The MS in the first 2 weeks of life appeared to have long-term effects on the stress response and subsequent visceral pain sensitivity in the pups. Independent researchers have reported similar heightened visceral sensitivity using these animal models (Coutinho et al., 2002; Ren et al., 2007; Moloney et al., 2012), with results indicating that female animals demonstrated greater sensitivity to MS compared to males.

#### Water Avoidance Stress

As the name suggests, animals were subjected to periods of water avoidance (WA) as a form of stress. In this model, male Wistar rats were subjected to either WA or sham WA for 1-h daily, 10 days sequentially. Previous data showed that rats subjected to chronic application of WAS for 10 days had visceral hyperalgesia which persisted for at least a month, increased rate of fecal pellet excretion, and evidence of low-grade immune activation such as expression of IL-1β and IFN-γ stress (Bradesi et al., 2005).

### *In vitro* Models for IBS

#### Human Gut-on-a-Chip

The innovation and development of microfluidic organ-on-a-chip models of human intestine have revolutionized the way in which we can study intestinal physiology and pathophysiology. Previously it was a great challenge to co-culture intestine microbes with viable epithelium for longer than 1 day using conventional culture models, and this was not achievable even with intestinal organoid cultures. Using a 2-D cell culture format with monolayer Caco-2 cells, for example, it is not possible to reproduce the physiological conditions present in the intestines, such as the unique intestinal tissue morphology of villi formation and mucus production, as well as key intestinal differentiated functions including cytochrome P-450-based drug metabolism. Moreover, the commensal bacteria will rapidly overgrow and contaminate the human cell cultures in these co-culture models. Recently, with the invention of microfluidic Organ Chip models of human intestine, these challenges have been overcome (Bein et al., 2018).

One of the earliest “human gut-on-a-chip” devices was developed by Kim and co-workers which was a revolutionary peristaltic, microfluidic, 2-channel Gut Chip model comprising a porous flexible membrane serving as a scaffold for intestinal epithelial cells and coated with ECM, that is sandwiched between 2 microfluidic channels. This microgut device mimics the dynamics (peristalsis), structure and physiological functions (absorption and transport) of human intestine and allows the growth of not only human intestinal cells but also capillary endothelial cells, immune cells, and even microorganisms (Kim H.J. et al., 2012). Interestingly, a normal intestinal microbe (*Lactobacillus rhamnosus* GG) was able to be co-cultured with the intestinal cells on the luminal surface for more than 1 week using this gut-on-a-chip. Huh et al. (2013) described the microengineering behind the fabrication of these organs-on-chips, detailing the materials and dimensions used to construct the structures of these microdevices, as well as the mechanical engineering principles that reproduce the peristalsis-like motions and fluid flow which closely mimic the real *in vivo* situation in the live intestine.

The microfluidic device was intended for drug permeability test, and pharmacokinetics (PK) and pharmacodynamics (PD) evaluation whereby the test drug could be delivered either to the upper or bottom channel via a pressure-driven flow to mimic dynamic *in vivo* conditions, but it could also be adapted to microbiome studies (Kimura et al., 2015). A notable application of the Gut Chip is to investigate the crosstalk between the immune system and the microbiome, a hallmark of chronic inflammatory diseases including IBD (Kim et al., 2016). It is foreseeable that applications of the Gut Chip will be extended to other disease models including IBS models in the future. A commercialized gut-on-a-chip system is the perfused gut epithelium tubules —-3D intestinal tubules in the OrganoPlate^®^ (MIMETAS, 2018).^1^

### Methodologies for Studying Microbiome

#### Fecal Sample Collection

The last few years had seen the formation of research consortia to investigate the human gut microbiome through metagenome analysis (Qin et al., 2010). These large-scale projects need efficient techniques and standardized methods for sample collection and processing to ensure accurate analysis and interpretation. In particular, DNA extraction from fecal/intestinal samples is one of the crucial steps in determining the composition of microbiota flora. Besides the presence of certain inhibitors from fecal samples, the differences in morphological and physicochemical properties, as well as the phylogenetic diversity will affect DNA extraction and subsequent downstream analysis. A number of studies had compared different sample collection methods, and found that fecal occult blood test card and RNAlater were deemed to be more stable without freezing for 4 days, but if the samples had to be frozen, then swab, FOBT, and 70% ethanol gave the highest accuracy (Sinha et al., 2016; Vogtmann et al., 2017; Wu et al., 2018).

#### Culture-Independent Approach

Besides that, different approaches have been developed to study the microbiome, including clone libraries, terminal restriction fragment length polymorphism (T-RFLP), quantitative PCR (qPCR), transcriptome microarrays, as well as high throughput sequencing technologies (Illumina sequencing and 454 pyrosequencing). Notably, pyrosequencing of 16S rRNA amplicons and Illumina sequencing are two robust methods for investigating the genome diversity and the differential gene expression of microbial communities in human guts (Qin et al., 2010) and oral cavities (Lazarevic et al., 2009). These technologies not only accelerated metatranscriptomic studies of microbiome, but together with metagenomic data sets, they also provide a great opportunity for exploring the structure and function of microbial communities. Moreover, these methods are culture-independent, allowing further studies and the identification of non-culturable microorganisms in the microbiome.

#### Culturomics

Nevertheless, one major limitation of metagenomic studies is the depth bias as current metagenomics technologies are unable to detect bacteria at concentrations of < 10^5^ bacteria per gram of stools. Although metagenomics techniques are able to yield the DNA sequences of many “uncultivable” bacteria, we still require viable microorganisms in pure culture for downstream applications such as for development of microbiota-based therapeutics. Under this circumstance, “Culturomics,” which is diversification of culture conditions together with identification by matrix-assisted laser desorption ionization–time of flight mass spectrometry [MALDI-TOF MS], is a set of methodologies aimed at increasing the bacterial repertoire from a microbiota flora. Cultured isolates will not only expand the reference genome databases, they may also reveal novel genes/functions for development of new therapeutics. A landmark culturomics study by Lagier and coworkers which utilized 212 different culture conditions in conjunction with MALDI-TOF-MS for identification, have produced 340 species of bacteria, 5 fungi and 1 giant virus from three stool samples (Lagier et al., 2012). This had led to the rebirth of culture techniques and the development of taxogenomics for classification of new bacteria (Lagier et al., 2015).

#### Metabolomics

The gut microbiota is the chief “organ” responsible for some crucial metabolic functions that include biosynthesis of amino acids, short chain fatty acids, essential vitamins (e.g. K and B12), bile acid biotransformation and hydrolysis as well as fermentation of non-digestible polysaccharides (Putignani et al., 2014). Therefore, metabolomics, which is the large-scale study of small molecule metabolic products (the metabolome) of cells, tissues, biofluids and organisms at a specific point in time; is a powerful approach to unravel the complex interactions of the metabolites from gut microbiota (metabolome). Comparative studies of metabolome for unhealthy and healthy subjects’ microbiota can lead to discovery of unique metabolites that could be developed as diagnostic or prognostic biomarkers. Two principal technologies that can be exploited for metabolomics are mass spectrometry (MS) and nuclear magnetic resonance (NMR) spectroscopy as these offer a good range of coverage, sensitivity and quantification (Vernochi et al., 2016).

Using metabolomics approach, a previous study demonstrated that the metabolite profile of fecal extracts from ulcerative colitis patients with and without IBS had alterations compared to healthy controls (Le Gall et al., 2011). It was also found that perturbed microbial metabolism results in an increased production of hydrogen gas in IBS, as well as indole, phenols and other compounds (Kumar et al., 2010). Volatile organic compounds (VOCs) are released by bacteria as by-products of metabolism, which could be determined by SPME-GC-MS. These specific microbial VOCs profiles could potentially serve as specific biomarker candidates for diagnostic purposes (Bunge et al., 2008). Overall, the current approaches and their applications in studying microbiome is summarized in Table 2.

**Table 2:** Current methodologies for studying microbiome.

Approach	Examples	Advantages/Features
Culture-independent approach	• Clone libraries • terminal restriction fragment length polymorphism (T-RFLP) • quantitative PCR (qPCR), • transcriptome microarrays, • high throughput sequencing technologies • metagenomics	(1) Enables the discovery of non-cultivable species in the gut (2) Unravel the structure and function of microbial communities (3) Untangle the genome diversity and the differential gene expression of microbial communities (4) Identification of the role of microbes in disease development

Culture-dependent approach	Culturomics (different culture conditions, with identification performed via microbiological methods as well as MALDI-TOF MS)	(1) Enables the recovery of microbes from the samples for downstream applications (2) Expansion of available reference genome databases (3) Potential approach to discover novel genes/functions for development of new therapeutics

Metabolomics	Mass Spectrometry (MS) and Nuclear Magnetic Resonance (NMR) Spectroscopy	(1) Enables the identification of metabolic products at specific time interval (2) Unravel the complexity between metabolites and gut microbiome (3) Enables the discovery of unique metabolic signature for diagnostic/prognostic applications

## IBS Therapy Options

Nowadays, a great myriad of therapeutic options are available for IBS. However, treatment outcome is still unsatisfactory for both the patient and doctor (Moayyedi et al., 2017). An important consequence of the associated comorbidities and treatment failure of IBS is reduced quality of life, and decreased work productivity. To illustrate this point specifically, IBS patients were found to be absent from work 2 days/month on average, with reduced work productivity for 9 days/month, according to a report (Buono et al., 2017).

**FIGURE 2:**
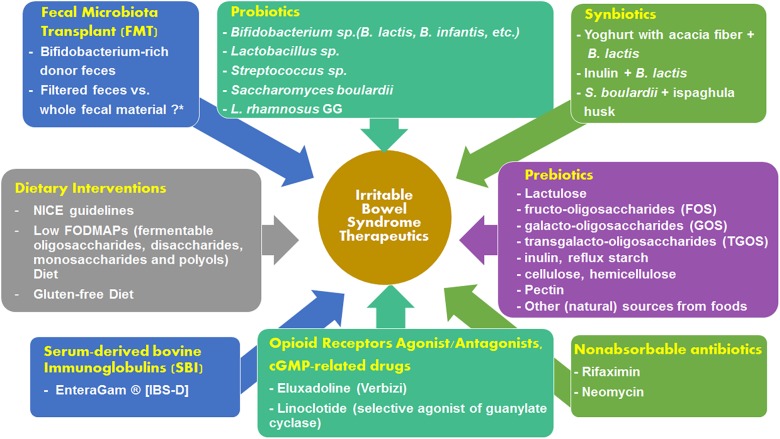
Summary of the available treatment options for mitigating the severity of IBS symptoms.

The fecal microbiota of IBS patients has been known to exhibit several qualitative and quantitative alterations (Dupont, 2014). There has also been a theory of microbial etiology which pinpoints bacterial, viral and parasitic infections for causing IBS (Klem et al., 2017). Therefore, targeting the microbiome in IBS could be an effective therapeutic approach. A subset of IBS patients show good response to non-absorbable antibiotics (Pimentel et al., 2011) and prebiotic/probiotic administration (Moraes-Filho and Quigley, 2015). Fecal transplantation is a new mode of IBS treatment which has gained traction and is lauded as a very promising therapeutic option in the near future. Figure 2 summarizes the therapeutic options available for alleviating the symptoms of IBS. The various treatment options are discussed in the sections below.

### Microbiota-Based Therapies: Prebiotics, Probiotics, and Synbiotics

#### Probiotics

Probiotics are living microorganisms which commonly comprises gut-friendly bacteria and sometimes also yeast, and are ingested in the form of foodstuffs and supplements. With the known pathophysiology of IBS, consistent use of probiotics has been demonstrated in previous studies to improve symptoms associated with IBS particularly with the *Biofidobacterium* and *Lactobacillus* strains (Guyonnet et al., 2007; Simrén et al., 2013). Several meta-analyses of randomized controlled trials (RCTs) that compared the effects of probiotics against placebo in reducing IBS symptoms (Ford et al., 2014; Didari et al., 2015; Zhang et al., 2016) found probiotics to be more superior to placebo in reducing overall IBS symptoms and abdominal pain. These meta-analyses looked at single probiotic strains, but other meta-analyses had examined the effects of combinations of strains *Bifidobacterium*, *Lactobacillus*, and *Streptococcus* genera (Jafari et al., 2014). Meta-analyses with less common probiotic genera or species were also conducted, such as with the yeast *Saccharomyces boulardii* (McFarland, 2010), bacteria *B. infantis* (Yuan et al., 2017) and *Lactobacillus rhamnosus* GG (Horvath et al., 2011) albeit with differing results. *S. boulardii* was reportedly able to lead to increase in bowel frequency, whereas *L. rhamnosus* reduced the intensity and frequency of abdominal pain. *B. infantis* in combination probiotics was able to reduce IBS symptoms (bloating and abdominal pain), but not *B. infantis* alone (Yuan et al., 2017).

Although in general terms, probiotics seem to have beneficial effects on improving IBS, how they function is still relatively unknown. Previous studies that attempted to illuminate their mode of action suggest that the probiotics strains played a role in modulating gut inflammation, producing antimicrobial peptides that help to eliminate pathogenic bacteria, and improving the mucosal barrier function. Much remains to be answered as to which strains is/are most effective across the broad spectrum of IBS patients and whether single or combination of strains work best. At the moment it seems to be a case of trial and error. This leads to the question of whether individual differences among IBS patients in terms of immune profile and microbiome diversity could affect the efficacy of probiotics therapy. Personalized or customized probiotics therapy guided by individual microbiota profiling may be the way forward in future.

#### Prebiotics

A disadvantageous feature of probiotics is that most have a short lifespan and thus repeated doses are necessary. Prebiotics may serve as an alternative treatment as they provide the metabolizable substrates for growth of specific bacteria and hence can alter the microbiota. According to the Food and Agriculture Organization of the United Nations (FAO), prebiotics are defined as non-viable food components that improve host gut health via altering the microbiota (Pineiro et al., 2008). One of the earliest synthetic prebiotics developed is lactulose, which is shown to increase gut bacteria, enhance water retention in stools, and is thus associated with laxative effects. Other prebiotics include fructo-oligosaccharides (FOS), soybean oligosaccharides, galacto-oligosaccharides (GOS), isomalto-oligosaccharides, xylo-oligosaccharides, and transgalacto-oligosaccharides (TGOS). The fructan inulin, cellulose, hemicellulose, reflux starch, and pectin are polysaccharide prebiotics. Many sources of prebiotics exist in nature including cereals, fruits and vegetables. Lactulose, lactosaccharose, FOS, GOS, and cyclodextrins are artificially synthesized prebiotics that can be commonly found as food additives or components in food production.

The benefits of prebiotics to gut health are multi-pronged. Commensal bacteria in the colon can ferment prebiotics to produce short chain fatty acids (SCFAs) such as acetate, butyrate and propionate. For instance, most strains of *Bifidobacterium* and *Lactobacillus* can utilize FOS (Kaplan and Hutkins, 2000). In fact, recent data showed that different *Bifidobacterium* strains have complementary pathways for utilizing the prebiotics inulin-type fructans and arabinoxylan oligosaccharides (AXOS) (Rivière et al., 2018). The SCFAs produced as metabolic end-products of prebiotics fermentation are capable to bind to GPR43, GPR41 and GPR109A, ‘metabolite-sensing’ G-protein coupled receptors which regulate the inflammatory responses, thereby affecting gut homeostasis. Moreover, prebiotics can help to correct dysbiosis by promoting positive alterations in the microbial flora, for instance enhancing the proliferation of gut bacteria including *Bifidobacterium* (Paineau et al., 2008; Silk et al., 2009). Prebiotics can also regulate cholesterol biosynthesis and lipid production in the host, as well as satiety (Rivière et al., 2016).

#### Synbiotics

As its name implies, synbiotics refer to the combination of probiotics and prebiotics in food ingredients or supplements in a form of synergism. In theory, synbiotics should be more potent or efficacious than their probiotics or prebiotics components used in singularity. However, the relatively limited number of clinical trials on synbiotic therapies in IBS patients have thus far produced varying results which could be due to the different probiotic and prebiotic components used in different trials, as well as the different subsets of IBS patients studied (Harris and Baffy, 2017).

It is interesting to note that an open, uncontrolled, multicenter study involving 43 centers focused on 636 IBS constipation-predominant patients who were given a synbiotic composed of *Bifidobacterium longum* W11 and the short chain oligosaccharide prebiotic Fos Actilight, had reported significant improvements in stool frequency, bloating and abdominal pain (Colecchia et al., 2006). One randomized controlled study found that a synbiotic consisting of a mix of 29 soil-based microbes with a prebiotic, leonardite (a complex of humic substances) had resulted in significant reductions in IBS symptoms compared to placebo. Nonetheless, >80% of patients had remission of IBS at the 52-week follow-up (Bittner et al., 2005). Another study on 130 IBS patients revealed that a synbiotic of composite yogurt enriched with acacia fiber and *Bifidobacterium lactis* was associated with significant improvement on IBS symptoms and bowel habit vs. a placebo yogurt (Min et al., 2012). Moreover, Baştürk and coworkers found that a *B. lactis* B94 and 900 mg inulin synbiotic had more pronounced efficacy in reducing belching-abdominal fullness, bloating after meals, constipation and mucus in the feces than the synbiotic alone in IBS children (Baştürk et al., 2016). On the contrary, Abbas and colleagues found that a 6-week course of *S. boulardii* plus ispaghula husk combination therapy did not improve overall symptom severity scores in IBS-D patients; although it had led to diminished proinflammatory cytokines interleukin-8 and TNF-α, and an increase in the anti-inflammatory cytokine IL-10 (Abbas et al., 2014). Clearly, conflicting results from the limited studies thus far warrant the need for more RCTs to prove the virtues of synbiotics in managing IBS.

### Non-absorbable Antibiotics

Several large-scale treatment trials and meta-analyses have shown that non-absorbable antibiotics such as Rifaximin and neomycin are effective for treatment of IBS. An antibiotic with a broad range of antibacterial effects against aerobic and anaerobic organisms that inhabit the GI tract, Rifaximin (Xifaxan^®^, Salix Pharmaceuticals, Bridgewater, NJ, United States) is a rifamycin derivative that was approved by the US Food and Drug Administration in 2015 for the treatment of IBS-D in adults. It is interesting to note that less than 0.5% of the oral dose is absorbed. Because it is poorly absorbed, rifaximin has low toxicity, insignificant adverse effects and drug interactions (Saadi and McCallum, 2013).

Three multicenter double-blind, placebo-controlled, phase 3 trials (TARGET 1–3), had demonstrated the efficacy and safety of rifaximin in improving the symptoms of IBS. The symptom relief was defined as a decrease of at least 30% from baseline in weekly IBS-related abdominal pain or discomfort and a weekly stool consistency score of less than 4 (Pimentel, 2018). TARGET 1 and TARGET 2 trials on IBS patients without constipation had resulted in global symptom relief for at least 2 weeks within a treatment-free month. The patients had received either rifaximin (550 mg) or placebo three times daily for a 2-week duration; and were subsequently subjected to a 10-week observation period (Pimentel et al., 2011). In the TARGET 3 trial, patients who had relapse of IBS-D after a short, 2-week duration first treatment were given repeat treatment of rifaximin. A significantly higher number of patients who were treated again with rifaximin vs. placebo were responders for abdominal pain but not stool consistency (Lembo A. et al., 2016). From this study, it was also shown that the repeat treatment was able to prevent recurrence besides prolonging the period of IBS symptom relief. Previous studies suggest that subpopulations of IBS patients have overgrowth of bacteria in the small bowel, which is known as small intestinal bacterial overgrowth (SIBO) (Giamarellos-Bourboulis et al., 2016). In patients with SIBO, rifaximin treatment had caused remarkable improvements from baseline in IBS symptoms (Meyrat et al., 2012). On the other hand, another antibiotic, neomycin has been shown to improve global IBS symptoms by 50%. Nonetheless, unlike rifaximin, neomycin had adverse effects and induced rapid bacterial resistance or *Clostridium difficile* infection (Distrutti et al., 2016).

### Opioid Receptors Agonist/Antagonists and cGMP-Related Drugs

Eluxadoline (Viberzi, Allergan) is a drug having mixed effects against opioid receptors (mixed μ-opioid receptor agonist–δ-opioid receptor antagonist and κ-opioid receptor agonist). Two phase 3 trials which compared 70 and 100 mg twice-daily doses of eluxadoline indicated that the higher dose brought about reduction of overall IBS symptoms, and improved quality of life compared to placebo (Lembo A.J. et al., 2016). The efficacy of treatment effects of eluxadoline on relief of IBS symptoms was on par with rifaximin.

Nonetheless, eluxadoline is associated with side effects. Common side effects from the two phase 3 trials were constipation, abdominal pain, nausea, vomiting, abdominal bloating, and gastroenteritis. Long term usage was cautioned due to the occurrence of serious adverse events (SAEs) linked to eluxadoline use in 4% of the patients (Cash, 2016). From 2015 until 2017, 120 cases of pancreatitis were reported. In 2017, a Drug Safety Communication was issued by the FDA about an association between eluxadoline use in patients without a gallbladder and increased risk of severe pancreatitis that could be fatal.

Linaclotide (Constella^®^, Allergan Inc.) is a selective guanylate cyclase C (GC-C) agonist which is efficacious in treating constipation-predominant IBS, based on latest meta-analysis of three phase III trials specific for IBS-C (Chey et al., 2012; Rao et al., 2012; Shah et al., 2018). Plecanatide is another GC-C agonist that was found to be effective for IBS-C management (Shah et al., 2018). In a mouse model, linaclotide binds to GC-C receptor expressed on the luminal surface of intestinal epithelial cells, which leads to stimulation of cyclic guanosine monophosphate (cGMP) release. The cGMP inhibits colonic noniceptors and acts as a second messenger in the downstream facilitation of intestinal fluid secretion (Castro et al., 2013). GC-C receptor and its effectors can also modulate intestinal fluid homeostasis and afferent gut nerve activity (Busby et al., 2010). These could be the underlying mechanisms for the ability of linaclotide to reduce abdominal pain in IBS-C patients.

### Serum-Derived Bovine Immunoglobulins

Serum-derived bovine immunoglobulin (SBI), marketed under the brand name EnteraGam^®^, has been approved by the US Food and Drug Administration for use in patients with chronic loose and frequent stools including IBS-D and IBD under physician supervision (EnteraGam Product Information Sheet, 2015). The EnteraGam SBI is essentially a formulation containing > 90% protein, with > 50% being immunoglobulin G (IgG) which is the active ingredient. In general, SBI had been shown by various small open-label studies to improve GI symptoms (i.e., stools frequency per day, ease of passage, and sense of evacuation). The mechanistic basis for the improvement of symptoms was investigated in a study and the findings showed that SBI therapy had led to alterations in the microbiome, particularly for Proteobacteria *Burkholderiales* and Firmicutes *Catonella* in the duodenum brushings but not in the stools. The SBI treatment had also not resulted in any changes in intestinal permeability nor bile acid synthesis. Hence the underlying mechanism for SBI’s benefit toward IBS-D patients is still unknown (Valentin et al., 2017).

A pilot study on a small number of subjects had shown the efficacy of SBI in IBS-D treatment (Wilson et al., 2013). A recent study on a total of 1,377 patients with IBS or IBD based on a one-page survey found that a remarkable number of patients had their stool frequency normalized to less than or equal to 4 times/day. The study assumed that the patients with IBS had IBS-D since SBI was prescribed mainly for IBS patients with IBS-D (Shaw et al., 2017).

### Dietary Interventions

Dietary interventions have been helpful for IBS patients as most of them report aggravation of symptoms associated with the ingestion of certain foods. In fact, modification or restriction of dietary intake in IBS patients had been implemented to improve or prevent gastrointestinal symptoms (Hayes et al., 2014). Two such diets are the FODMAP restriction and the gluten-free diet.

#### Low-FODMAPs Diet

FODMAP is an acronym for Fermentable – Oligosaccharides – Disaccharides – Monosaccharides – And – Polyols. Briefly, FODMAPs consist of short chain carbohydrates such as fructans, polyols and galacto-oligosaccharides that are poorly absorbed in the small intestine (Gibson and Shepherd, 2005). Fructose and lactose are also on the FODMAPs list as the absorption mechanism (in the case of fructose) or the enzyme responsible for breaking down the sugar (lactose) are impaired in IBS patients. Consumption of FODMAPs are closely linked with IBS development (Staudacher et al., 2012; Böhn et al., 2015). There are many randomized, controlled trials in the literature which reported the effectiveness of FODMAPs restriction diet in improving global IBS symptoms, visceral pain, bloating and quality of life (QOL) in >50% of IBS sufferers (Dolan et al., 2018). According to Britain’s National Institute for Health and Care Excellence (NICE) guidelines for dietary and lifestyle advice, dietary and nutritional perspectives should be considered in administering appropriate advice to IBS patients. Particular attention was given to limitation on intake of insoluble fibers and starch, with ispaghula powder (a soluble fiber) or foods high in soluble fiber being encouraged. The NICE guidelines recommend a more restrictive diet such as low FODMAPs if the IBS symptoms still persist after following the general dietary guidelines (National Institute for Health and Care Excellence [NICE], 2017).

A meta-analysis on the low FODMAP diet based on six RCTs on a total of 182 patients and 172 controls demonstrated an improvement in severity of IBS symptoms and QOL scores (Marsh et al., 2016). Interestingly, a recent paper on a randomized, controlled crossover study showed that a low-FODMAP diet was more superior to a standard Australian diet in improving global IBS symptom scores. Improvement in stool consistency was seen in all patients regardless of IBS subtypes although stool frequency was improved only in IBS-D patients (Halmos et al., 2014).

In a recent meta-analysis, Varjú et al. (2017) conducted pooled analysis from 10 studies comprising RCTs, non-randomized controlled trials and non-controlled prospective trials. A standardized complex outcome score, the IBS-Symptom Severity Score (IBS-SSS) was used for the endpoint scoring. This meta-analysis proved statistically that a low-FODMAP diet does have beneficial effect on IBS-SSS, as compared to a high-FODMAP standard IBS diet recommended by the guidelines (Varjú et al., 2017). Nonetheless, in a more updated systematic review and meta-analysis, Dionne and coworkers looked at the effects of a gluten-free diet (GFD) and a low FODMAPS diet in alleviating the symptoms of IBS (Dionne et al., 2018). Patients on GFD had reduced global symptoms compared with those on a control diet, although this was not statistically significant. On the other hand, there is evidence that a low FODMAPs diet could help in reducing global IBS symptoms (Dionne et al., 2018).

Despite its potential positive value, a low FODMAPs diet is associated with limitations particularly with regards to its highly restrictive nature, its possible adverse effects on nutritional status in the long term, and the importance of close monitoring by dieticians. A low FODMAPs diet also poses the danger of reducing the abundance of commensal bacteria. A recent study had shown that fecal bacteria such as *Actinobacteria*, *Bifidobacterium*, and *Faecalibacterium prausnitzii*, in patients on a low FODMAPs diet were reduced significantly along with reduction in pro-inflammatory cytokines (Hustoft et al., 2017). Further studies with more defined patient cohorts and symptoms as well as clear and standardized measurements of IBS symptoms pre- and post-intervention should be conducted to investigate the overall effect of a low FODMAPs diet compared to traditional IBS diet.

#### Gluten-Free Diet

Gluten, a protein found in grains such as wheat, rye, spelt and barley has been touted as the culprit for a wide range of gastrointestinal related disorders. Several studies had investigated the efficacy of a gluten-free diet (GFD) in reducing IBS-related symptoms. Vazzquez-Roque and coworkers conducted a 4-week RCT of gluten-free diet in 23 IBS-D patients who were further subgrouped according to their HLA genotype (12 HLA-DQ2/8-negative and 11 HLA-DQ2/8–positive), with 22 patients in the control group on gluten-containing diet. They found that gluten-free diet had impacted the stool frequency, with statistically significant decrease in stool frequency in individuals on a GFD versus subjects on a gluten-containing diet, and the effect was more pronounced in HLA-DQ2 or 8 positive patients than HLA-DQ2/8 negative patients. Gluten-containing diet was associated with higher small bowel mucosal permeability which was measured using urine sugars excretion as biomarkers (Vazquez-Roque et al., 2013).

In another study, 41 patients with IBS-D who were either HLA-DQ2/8-positive or -negative were placed on a 6-week GFD. The GFD was associated with markedly reduced IBS-SSS score in 71% of patients in total. In the study, HLA-DQ2/8-positive subjects reportedly had greater reduction in depression as well as in vitality score than HLA-DQ2/8-negative subjects (Aziz et al., 2016). The observation that HLA-DQ genotype had a bearing on usefulness of gluten-free diet in improving IBS symptoms suggests that an adaptive immune response may play a role in gluten’s effects on gut barrier function. Furthermore, there is speculation that an innate immune response could also be involved, since toll-like receptors (TLR) expression is increased in mucosa of gluten-sensitive patients.

#### Fecal Microbiota Transplant

Strong evidence for the role of dysbiosis in IBS pathogenesis has elicited the proposition to cure IBS patients through fecal microbiota transplantation (FMT). FMT is an approach which involves the application of a solution of fecal material from a healthy donor into the gut of a receiver, with the intention of restoring the aberrant microbial composition in the gut to a healthy homeostasis. Treatment via FMT had become popular in the past 5 years but in fact, this therapeutic approach has been long practiced since the 4th century in ancient China, by a physician called Ge Hong, whereby he had suggested to patients with severe diarrhea to use fresh stool as a choice of treatment (Rossen et al., 2015).

Numerous studies have clearly linked abnormal gut microbiota composition with IBS development (King et al., 1998; Malinen et al., 2005; Kassinen et al., 2007). Nevertheless, these studies also questioned the suitability of FMT as a form of treatment against IBS. This is owing to the lack of understanding on the microbial pathophysiology. Furthermore, there is no robust, definitive conclusion regarding the exact mechanism underlying IBS pathogenesis, whether IBS is caused by changes in the gut microbiota composition or due to a consequence of the alteration of intestinal secretion and motility.

Based on a systematic review conducted by Halkjær et al. (2017) on IBS and FMT, the authors reported that 28 out of 48 patients (58%) experienced positive outcomes after FMT treatments. Moreover, there were no serious adverse events reported. Meanwhile, in another study by Mizuno et al. (2017) in Japan, the authors revealed that FMT treatment had improved the psychological status and stool form of IBS patients. Additionally, the authors showed that presence of *Bifidobacterium*-rich donor feces could be a promising indicator for successful FMT treatment in IBS. The authors speculate that *Bifidobacterium*-rich feces trigger the minor strains formation of the microbiota growth which could help to increase the diversity after FMT treatment. In another study, it was demonstrated that the effect of using oral fecal capsules was comparable to colonoscopy-administered transplantation in alleviation of IBS symptoms (Kao et al., 2017).

Johnsen et al. (2017) had assessed the effect of FMT on moderate-to-severe IBS-D and IBS-M patients through a randomized, double-blind and placebo-controlled trial. The authors demonstrated that a significant number of patients in the active treatment group versus the placebo group (65% vs. 43%) responded well to the treatment and had reduced IBS symptom severity after 3 months. However, the severity was not alleviated after 12 months. These results serve as an important breakthrough in evaluating the efficacy of FMT treatment in IBS. Nevertheless, larger multicenter studies are warranted to confirm these findings. The efficacy of FMT is still debatable at this stage, owing to newer findings that filtered feces could have the similar effects as whole fecal material transplantation (Ott et al., 2017). This raises the concern of whether bacteriophages and postbiotics, or metabolites secreted by the microbiota are actually the effective agents as these substances are able to get through the filters. More studies are in demand to confirm the actual mechanism and beneficial effect of FMT and the therapeutic agents responsible for the success of FMT treatment in IBS.

## Strategies for Future IBS Therapeutics

**FIGURE 3:**
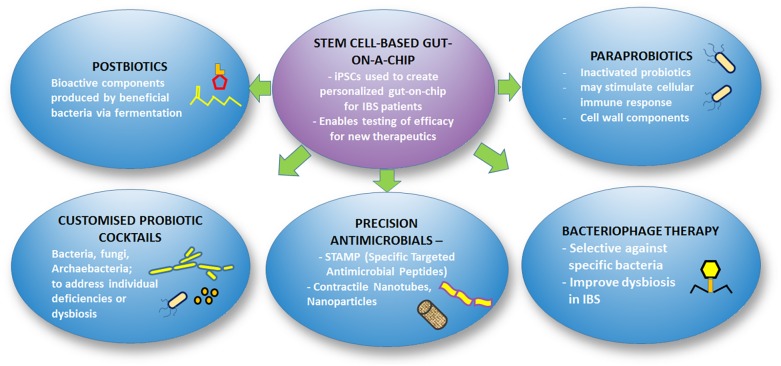
Overview of future therapeutics for IBS.

As our understanding of the microbiome gradually unravels, the potential to manipulate the microbiome for improvement in health and disease treatment is widening. As the costs of metagenomics sequencing become more affordable, the ability to obtain the microbiome profile of individuals for diagnostic purposes has been made possible. Along with this, soon we might be able to determine the exact profile of microbiota dysbiosis in each patient and devise personalized treatment such as customized probiotic formulations, targeted antimicrobials or immunotherapeutics for the individual patients. Figure 3 illustrates the framework of future therapeutic strategies for IBS.

### Stem Cell-Based Gut-on-a-Chip

A stem cell-based gut-on-a-chip had been created with cutting-edge technology and are virtually miniature versions of a human intestinal lining, termed organoids, derived from induced pluripotent stem cells (iPSCs). The iPSCs were derived from skin or blood samples collected from a patient and reprogrammed into intestinal villi stem cells (Workman et al., 2018). The gut-on-a-chip is combined with microfluidic engineering to mimic the dynamic microenvironment around the gut cells. By creating a personalized gut-on-a-chip for selected IBS patients, we can gain valuable understanding on the efficacy of new drugs on individual patients, instead of subjecting a patient to needless possible side effects. Emulate, a company that specializes on development of organ-on-chips including Intestine-Chip for toxicology testing, was born out of a commercialization effort of the founding team that created the organ-on-chips^2^.

### Microbiome-Based Future Therapeutics

#### Bacteriophage Therapy

Bacteriophages (viruses that infect bacteria) are ubiquitous, naturally occurring entities that control microbial populations and have an important role in controlling the microbial communities in the GI tract. From a metagenomics analysis, an estimated 1,200 viral genotypes, mostly siphophages and prophages within bacterial genomes in fecal contents were reported in the intestines of healthy humans (Mills et al., 2013). Changes in bacteriophage diversity have been reported in the diseased state, but different studies have shown contradictory results where some studies reported a decrease in diversity versus other studies which stated an enhanced diversity (McCarville et al., 2016). Since bacteriophages can exert selective pressure on targeted members of the bacterial community in intestinal microbiome, they may be manipulated for developing IBS therapies. Potential applications of bacteriophages include designing phage to “correct” microbiota dysbiosis, creating phage therapies to target certain bacterial species that causes a gut disease, and developing compounds that block phage induction to inhibit the growth of certain bacteria that requires phage to remain viable. In February 2018, the FDA has approved a phase 1/2a study of bacteriophages specifically designed against adherent-invasive *Escherichia coli* (AIEC), to treat IBD. More studies are needed for probing the possibility of including bacteriophage therapy among the limited arsenal of IBS therapy options available.

#### Postbiotics, Paraprobiotics, and New Probiotics Formulations

Recent studies have shown that live bacteria are not required for achieving beneficial effects when utilizing probiotics in treating symptoms of gut disorders. New names such as paraprobiotic and postbiotic have been minted to represent these non-living components of the microbiome. Postbiotics and parabiotics have biological activity and provide benefits to the host (Tsilingiri and Rescigno, 2013). Postbiotics are non-viable soluble factors (such as bacterial cell wall components, enzymes, peptides like glutathione, polysaccharides, organic acids, and short chain fatty acids) secreted by live bacteria or released upon cell lysis (Cicenia et al., 2014; Aguilar-Toalá et al., 2018). Other names for postbiotics are metabiotics, biogenics, or metabolites or cell-free supernatants. On the other hand, paraprobiotics, also known as “non-viable probiotics,” “inactivated probiotics” or “ghost probiotics,” refer to inactivated (non-viable) microbial cells that provide health benefits when given in appropriate doses (Taverniti and Guglielmetti, 2011; Tsilingiri and Rescigno, 2013). The advantage of postbiotics lies in its safety profile and longer shelf-life versus probiotics while conferring health benefits that are comparable to those of probiotics (Shenderov, 2013). Indeed, an interesting study by Tsilingiri and co-workers found that a postbiotic was more superior to a *Lactobacillus* probiotic in protecting the intestine (in this case an organ culture system of IBD model was used) against inflammatory response resulting from *Salmonella* invasion. This study suggested that live microorganisms in probiotics may not always be beneficial as it was found that certain probiotics could elicit a local inflammatory response, and hence postbiotics may be a safer alternative (Tsilingiri et al., 2012). In a recent study, it was shown that *L. casei* DG and its postbiotic attenuated the inflammatory mucosal response in an *ex vivo* organ culture model of post-infection IBS-D (Compare et al., 2017). Nonetheless, more studies are required to validate the beneficial effects of postbiotics either alone or in combination with probiotics for IBS treatment.

As the fungal and archaebacterial components of the gut microbiota as well as their role in maintaining gut health becomes clearer through new culturomics and metabolomics studies, it may be beneficial to formulate new probiotic cocktails that include these microorganisms. Theoretically, it may be possible to also design recombinant probiotics using bioengineering procedures to combine the beneficial attributes of specific bacteria or fungi.

### Precision Antimicrobial Compounds

Can biofilm-destroying compounds help to facilitate the antibiotics penetration into “resistant areas” or channel the antimicrobial to less accessible areas of the intestines and thus regulate these dysbiosis or imbalances? Or can one design a precision antimicrobial peptide that can specifically target a particular species *in situ*, while sparing the other members of the microbiota? One such example is the clever innovation of a specific targeted antimicrobial peptide (known as STAMP) which was designed by Eckert et al. (2006) to destroy *Streptococcus mutans* in a complex community of the oral biofilm. *Streptococcus mutans* is the chief culprit for dental caries. Similar strategies can be employed for modifying the gut microbiome dynamics and composition by targeting specific microbial species that has been identified to be associated with IBS symptoms. For example, in theory one could design precision antimicrobials or even use CRISPR-Cas9 technology to reduce the population of bacteria such as *Methanobrevibacter smithii* that produces methane for IBS-C patients who have methane breath.

Leveraging on the extensive database of peptides encoded by the gut microbes which are available freely on the MAHMI database^3^, the same group of researchers who developed the database had demonstrated that bacterial peptides FR-16 and LR-17 that are encoded by genes in *B. longum* DJ010A and *B. fragilis* YCH46, respectively, could modulate the immune response by elevating the Th17 and decreasing the Th1 cell response (Hidalgo-Cantabrana et al., 2017). Using bioinformatics *in silico* screening tools, we could screen the library of millions of peptides contained in the database to zoom in on manageable number of selected peptides for actual experimental studies to search for those with desired antimicrobial activity.

### Precision Nanotubes or Nanoparticles

Contractile nanotubes produced by certain bacteria, such as the R-type bacteriocins produced by *Pseudomonas aeruginosa*, as well as the contractile DNA injection systems produced by Myovirus bacteriophage T4, are naturally occurring precision antimicrobials. These contractile systems bind to receptors on bacterial cell surfaces, then inject a hollow tube into the cell wall, allowing ionic flux and depolarization of the inner membrane, thus killing the bacterial cell (Ge et al., 2015). Exploiting this unique property of contractile nanotubes, we can engineer nanotubes with different specificities to target microbes, by substituting the ligand-recognition domains of receptor-binding proteins from phages or from contractile bacteriocins.

## Conclusion

The global incidence of IBS is increasing as countries become more modernized. The pathogenesis of IBS is multifactorial, although consensus opinion within the medical profession holds that the gut microbiota plays a central role in disease development. Various existing and in-development treatment options are available but each IBS patient may require personalized treatment. It is believed that a holistic approach with a multi-disciplinary team of healthcare professionals that includes gastroenterologists, dieticians, clinical microbiologist and molecular genomics experts is required for effective diagnosis and management of IBS. IBS-C, IBS-M and IBS-D patients may require different treatment modalities. Currently there are still existing gaps of knowledge in terms of the state of dysbiosis in the microbiota in IBS and the pathophysiological mechanisms whereby targeted therapies can mitigate these imbalances. Further studies including large-scale, controlled trials of existing and new treatments are pertinent for enhancing our understanding in this field. Once these “missing links” have been uncovered, it is highly likely that precision medicine tailored for not only the different IBS subtypes but also for individual genetic and dietary differences can be engineered. In the imminent future, every patient may be able to get their complete genome and gut microbiome sequenced, as well as the gut metabolome data analyzed. These integrated multi-omic data will hopefully aid in decoding the microbiota community present in the patient, and serve as a guide for personalized treatment.

## Author Contributions

PC conceptualized and coordinated the work. PC, VC, CL, PM, WW, and VY contributed to this article in terms of data collection and writing. PC and VC performed critical revision for the work.

## Conflict of Interest Statement

The authors declare that the research was conducted in the absence of any commercial or financial relationships that could be construed as a potential conflict of interest.

## Footnotes

1 https://mimetas.com/overview-mimetas-applications/perfused-gut-epithelium-tubules

2 https://emulatebio.com/system/

3 www.mahmi.org
