# Corrigendum: The Microbiome and Irritable Bowel Syndrome – A Review on the Pathophysiology, Current Research and Future Therapy

**DOI:** 10.3389/fmicb.2019.01870

**Published:** 2019-08-13

**Authors:** Pei Pei Chong, Voon Kin Chin, Chung Yeng Looi, Won Fen Wong, Priya Madhavan, Voon Chen Yong

**Affiliations:** ^1^School of Biosciences, Taylor's University, Subang Jaya, Malaysia; ^2^Department of Medical Microbiology, Faculty of Medicine, University of Malaya, Kuala Lumpur, Malaysia; ^3^School of Medicine, Faculty of Health and Medical Sciences, Taylor's University, Subang Jaya, Malaysia

**Keywords:** irritable bowel syndrome, microbiome, microbiota dysbiosis, fecal transplant, IBS animal model

In the original article “Won Fen Wong” was incorrectly assigned as the second corresponding author. The sole corresponding author is “Pei Pei Chong.”

In addition, we neglected to include the funder “Taylor's University, TRGS/ERFS/2/2018/SBS/025” to Pei Pei Chong.

A correction has therefore been made to the **Funding** statement:

“This work was supported by the Taylor's Research Grant Scheme (TRGS), Taylor's University, Malaysia (Award No. TRGS/MFS/2/2016/SOM/013) and Taylor's University, (Award No. TRGS/ERFS/2/2018/SBS/025).”

The authors apologize for these errors and state that they do not change the scientific conclusions of the article in any way. The original article has been updated.

